# Fluoride release and mechanical properties of S-PRG fillers in dental materials: a systematic review and meta-analysis

**DOI:** 10.1038/s41405-026-00442-z

**Published:** 2026-05-15

**Authors:** Kanwalpreet Kaur, Ravinder S. Saini, Mohamed Saheer Kuruniyan, Rayan Ibrahim H. Binduhayyim, V.N.V. Madhav, Sunil Kumar Vaddamanu, Mario Alberto Alarcón-Sánchez, Artak Heboyan

**Affiliations:** 1 Rutgers School of Dental Medicine, Newark, NJ USA; 2 Department of Allied Dental Health Sciences COAMS, King Khalid University, Abha, Saudi Arabia; 3 Department of Prosthodontics, Crown & Bridge and Oral Implantology, Dr. D. Y. Patil Dental College and Hospital, Dr. D. Y. Patil Vidyapeeth (Deemed to be University), 411018, Maharashtra, Pune, India; 4 Molecular Biology and Medicine Program, University Center of Health Sciences, University of Guadalajara (CUCS-UdeG), Guadalajara, Jalisco Mexico; 5 Institute of Research in Dentistry, Department of Integral Dental Clinics, University Center of Health Sciences, University of Guadalajara (CUCS-UdeG), Guadalajara, Jalisco Mexico; 6 Department of Research Analytics, Saveetha Dental College and Hospitals, Saveetha Institute of Medical and Technical Sciences, Saveetha University, Chennai, India; 7 Department of Prosthodontics, Faculty of Stomatology, Yerevan State Medical University after Mkhitar Heratsi, Str. Koryun 2, Yerevan, Armenia; 8 Department of Prosthodontics, School of Dentistry, Tehran University of Medical Sciences, North Karegar St, Tehran, Iran

**Keywords:** Composite resin, Dental materials, Dental biomaterials

## Abstract

**Background:**

This systematic review aimed to assess the fluoride release and selected mechanical properties (direct tensile strength [DTS], flexural strength, and wear resistance) of S-PRG-containing dental materials.

**Method:**

A search was conducted using PubMed, Scopus, ScienceDirect, and Google Scholar according to the PRISMA guidelines. Qualitative data were pooled narratively, and quantitative data were synthesized statistically in RevMan 5.4 using a random-effects meta-analysis. Subgroup analyses of DTS and flexural strength were conducted where appropriate. The risk of bias was assessed using the QUIN tool, and GRADE was used to assess the certainty of the evidence.

**Results:**

Of the 216 studies identified, 18 were deemed eligible. Seven studies provided quantitative fluoride-release data for the meta-analysis. The meta-analysis revealed an overall statistically significant difference between S-PRG-containing materials and comparators (mean difference: −3.21; 95% CI: −4.12 to −2.30; *p* < 0.01) with very high heterogeneity between studies (I² = 98%). Subgroup meta-analyses of mechanical property comparisons revealed pooled estimates of 6.50 and 11.20 for the DTS and flexural strength comparisons, respectively. Both comparisons showed extreme heterogeneity between studies. Most studies demonstrated a moderate risk of bias, and the certainty of evidence was low.

**Conclusion:**

Owing to their composition, S-PRG-containing materials might have the potential for fluoride release and may have different mechanical properties compared to comparator materials. Nevertheless, there is insufficient evidence owing to the extreme heterogeneity and high number of in vitro studies. Therefore, the interpretation of these findings should be made with caution, and future investigations should use standardized testing protocols and well-designed clinical trials.

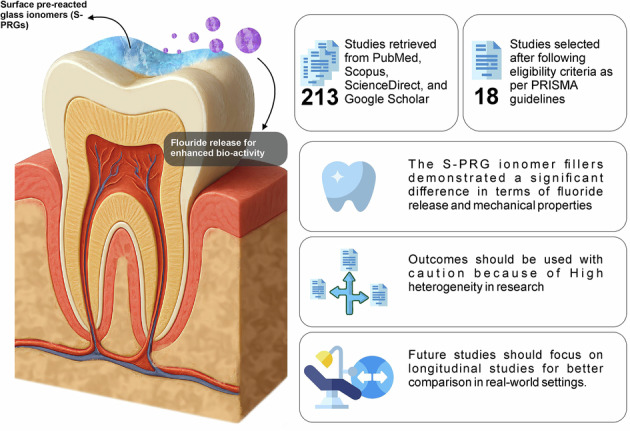

## Introduction

Oral health is a fundamental component of general well-being and health. Among oral diseases, dental caries has a significant burden on adults and children of all ages [1, 2]. The risk of dental caries has increased with frequent intake of sugars, which leads to an increase in the level of acid and causes disruption of the complex microbial community and symbiotic diversity [3]. This increase in acidity disrupts the symbiosis of the dental biofilm, ultimately leading to dysbiosis [4]. Meanwhile, the materials used for dental restorations are undergoing rapid advancement and mainly focus on two key components: ensuring long-term material performance and enhancing therapeutic benefits [5, 6]. Although dental materials used for restoration are designed to be durable and inert, they still degrade and deteriorate, and during the process, the constituents of these dental restorative materials are sometimes released into the oral cavity [7]. For instance, dental restoration materials, such as amalgam restorations, have a controversial role because of the hazardous mercury content [7]. In addition, silicate ceramics have poor mechanical properties compared with other restorative materials, such as resin-based and polycrystalline ceramics [8]. With the advent of glass ionomer cements (GICs), which are considered a cornerstone in dental restorations, one of the most beneficial properties is the release of fluoride [9]. Despite the biocompatibility of GICs, these materials still have clinical concerns, and their initial mixing can cause sensitivity and produce pulp irritation [10]. Furthermore, poor mechanical properties and high solubility are among the other limitations [11], which has led to the quest for the modification of GICs to enhance their strength and bioactivity.

Surface pre-reacted glass ionomers (S-PRG) represent a more advanced technological leap in this domain, and their production involves the melting of mixtures of mullite, silica, boric acid, strontium fluoride, cryolite, and strontium carbonate [12]. It can release multiple ions, such as fluoride, strontium, borate, sodium, aluminum, and silicate ions [13], which enhance the mineralization process and improve the biomechanics of demineralized dentin [14]. In particular, fluoride ions play a pivotal role in de-and remineralization by acting at the enamel-plaque contact, and their post-eruptive activity can lead to the development of innovative fluoride delivery systems [15]. In addition, S-PRG exhibits bioactive properties, such as acid neutralization, tooth strengthening, mineralization promotion, bacterial and fungal inhibition, cell enhancement, and inhibition of matrix metalloproteinases [13]. In vitro studies demonstrated the efficacy of S-PRG and found it to be effective in terms of inhibition of demineralization of the tooth substrate and also helpful in the prevention of caries development [16–19]. Furthermore, root canal sealers containing S-PRG have demonstrated good anti-inflammatory properties [20] and greater reliability and fracture loads than non-S-PRG fillers [21].

S-PRG is a promising bioactive restorative material with the potential for fluoride release and favorable mechanical performance. Its comparative performance remains dependent on the type of comparator material and testing conditions used across studies. S-PRG fillers are well known for releasing multiple ions, particularly fluoride ions, which are critical for the prevention of caries and maintenance of overall oral health. Despite its potential as a bioactive material with strength, there is still controversy in another study, and it was mentioned that the superiority, stronger strengthening bonds, and greater durability of S-PRG fillers have not yet been established [22]. Therefore, a comprehensive analysis is required to compare their efficacy and performance in terms of fluoride delivery systems, mechanical strength, and reliability. The findings of this review will be helpful in guiding material formulations and clinical applications to meet the demands of dental restoration procedures.

This systematic review aimed to evaluate the fluoride release, recharge capability, and mechanical properties of S-PRG-containing dental materials while considering the variation in comparator material class and laboratory testing methods across studies. In vivo evidence, such as a randomized clinical trial showing sustained fluoride retention in denture bases (Kiatsirirote et al., 2019) [23] and animal studies demonstrating the anti-inflammatory effects of S-PRG sealers (Miyaji et al., 2020) [20], supports its potential; however, such studies are limited due to ethical and logistical constraints, necessitating a focus on in vitro data.

## Materials and methods

This systematic review and meta-analysis was performed using the Preferred Reporting Items for Systematic Reviews and Meta-analyses (PRISMA) 27-item guidelines [24]. These guidelines are helpful for maintaining the quality, reporting, and transparency of reviews. This study was registered with the ***International Platform of Registered Systematic Review and Meta-analysis Protocols (INPLASY) under registration number 202540107***.

### Literature search strategy

For a comprehensive literature search (until September 2025), a systematic approach was followed using different electronic databases, including PubMed, Scopus, ScienceDirect, and Google Scholar. The searches yielded the following results: PubMed (45 hits), ScienceDirect (38 hits), Scopus (607 hits), and Google Scholar (320 hits). Keywords such as “surface pre-reacted glass-ionomer filler” OR “S-PRG fillers” AND “fluoride release” OR “fluoride delivery system” AND “mechanical properties” OR “strength” OR “flexural strength” OR “durability” OR “wear resistance” AND “dental restoration materials” OR “dental fillings” were used (see Supplementary Table 1).

### Eligibility criteria

For the selection of studies, the PICO guidelines were as follows: P (Population): Patients for dental restoration, I (Intervention): Use of S-PRG fillers for the treatment, C (Control/Comparator): placebo or non-S-PRG fillers, O (outcomes): flexural strength, wear resistance, durability, fluoride release, and performance. Randomized and non-randomized studies, such as cross-sectional, retrospective, observational, or in vitro studies, published in English in peer-reviewed journals were included. Similarly, studies that did not follow the PICO guidelines with incomplete data, reviews, editorials, letters to editors, commentaries, and case studies published in non-peer-reviewed and non-English journals were excluded.

### Selection of studies and data extraction

**Fig. 1:**
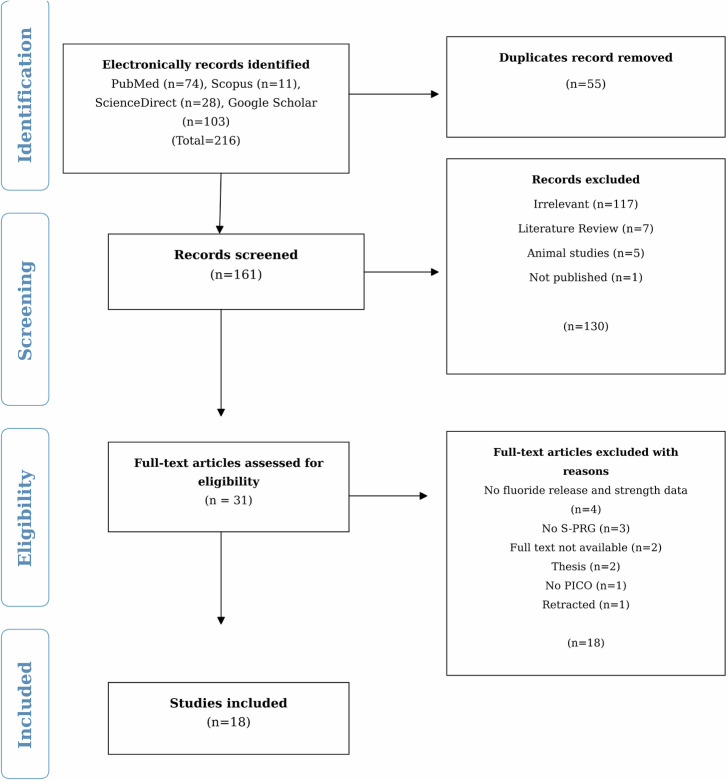
PRISMA flow diagram showing the identification, screening, eligibility assessment, and inclusion of studies in the systematic review and meta-analysis.

Two independent reviewers followed the four-stage PRISMA flowchart for study selection. In the first stage, 216 studies were identified and retrieved from different electronic databases and imported to the EndNote X9 referencing software, and 55 duplicate studies were excluded from the analysis. In the second stage, the remaining 161 studies were screened through titles and abstracts; 130 irrelevant studies were excluded, and the remaining 28 studies were selected for full-text assessment (3^rd^ stage). Each study was then evaluated according to the eligibility criteria, and 13 studies that did not meet the eligibility criteria were excluded for various reasons (Fig. 1). Finally, 18 studies were included and analyzed qualitatively and quantitatively. Disagreements during study selection were resolved through discussion, and when needed, a third senior reviewer was consulted for consensus. Likewise, two reviewers used a predefined data extraction form to independently recorded the study and outcome characteristics.

### Methodological quality assessment

The QUIN assessment tool, based on 12 items, was used for the methodological quality assessment of in vitro studies. Each item was rated as yes (1–2 points) or no/not applicable (0 points), according to the reviewers’ responses. Subsequently, a cumulative percentage for each study was calculated, and scores were mentioned and rated according to the following criteria: score <50% (high RoB), 50–70% (medium RoB), and >70% (low RoB) [25]. The Cochrane risk of bias-2.0 (RoB-2.0), based on five domains, was used for the quality assessment of randomized studies; each domain was categorized as low RoB, high RoB, and some concerns [26].

### Data analysis

A narrative synthesis was performed to summarize the study characteristics, intervention details, comparator materials, and outcome assessment methods. Quantitative synthesis was conducted in RevMan 5.4 (Cochrane, London, United Kingdom) using continuous data reported as sample size, mean, and standard deviation. To minimize transcription errors, quantitative data were extracted in duplicate from the included studies before the meta-analysis. For fluoride release, only studies reporting quantitatively compatible data were included in the meta-analysis. Studies reporting fluoride outcomes using non-comparable formats, incomplete dispersion measures, or only narrative/qualitative ion-release findings were excluded from quantitative pooling and summarized descriptively. This variation in outcome format also limited further stratification by the comparator class. Although fluoride release was commonly assessed using ion-selective electrode-based methods, variation remained in reporting format, immersion media, pH conditions, and measurement intervals, which was considered when interpreting heterogeneity.

Owing to the lack of chemical uniformity in the comparator groups and mixing of restorative material classes between conventional resin-based materials, glass ionomer-based materials, and other non-S-PRG materials, the pooled analysis was conservatively interpreted as an exploratory overall comparison. Subgroup meta-analysis according to the comparator class was not performed for fluoride release because the number of studies per control material category was small, and the reporting was insufficiently uniform to allow statistically robust stratification. Where sufficient homogeneity in reporting existed among the studies, subgroup analyses were performed for mechanical outcomes. Forest plots were used to display the effect sizes using a random-effects model. The I^2^ statistic was used to quantify the heterogeneity. I^2^ values were interpreted as low (<29%), moderate (30–49%), substantial (50–74%), and very high (>75%) heterogeneity. Tau-squared and Cochran’s Q test were used. A significance level of <0.01 was adopted [27, 28]. Differences in the fluoride measurement approach, reporting format, pH conditions, and comparator material class were considered likely contributors to heterogeneity.

### GRADE framework for certainty of evidence

The Grading of Recommendations, Assessment, Development, and Evaluation (GRADE) framework was used to assess the certainty of evidence in the domains of effect size, inconsistency, indirectness, and publication bias. After the assessment, the evidence was categorized as having very low, low, moderate, or high concern [29].

## Results

### General characteristics of studies

The general characteristics of the included studies are presented in Table 1. A total of 18 studies were included in this systematic review, with three additional studies (Ubolsa-Ard et al. [14]; Kaga et al. [30]; Islam et al. [22]) incorporated from updated searches to September 2025, as detailed in the PRISMA flow chart (Fig. 1). Most studies (14/18) were in vitro, focusing on controlled assessments of fluoride release and mechanical properties of S-PRG fillers, typically involving resin-based composites or sealants. Two studies were in vivo: Kiatsirirote et al. [23], a randomized clinical trial (n = 110, age: 49.7 years, 24 M:86 F), found that resin denture bases with S-PRG fillers (20% wt) increased salivary fluoride retention by 25% over 6 months compared with controls (*p* < 0.05). Miyaji et al. [20], in a rat model (*n* = 20/group), demonstrated that S-PRG root canal sealers reduced inflammation scores by 40% (histological analysis, *p* < 0.01) compared to silica-based fillers. The scarcity of in vivo studies is due to ethical constraints (e.g., invasive procedures in humans), high costs, and the need for preclinical in vitro validation to establish material safety and efficacy before clinical trials can be conducted. Studies were conducted in various countries, including Japan, the USA, China, Saudi Arabia, Romania, Turkey, Brazil, and South Korea. The sample sizes ranged from 3 to 60 specimens per group for in vitro studies, with one clinical trial involving 110 participants. The tested S-PRG materials included Beautifil II, BeautiSealant, and experimental resins, with compositions often comprising TEGDMA, UDMA, Bis-GMA, and S-PRG fillers (20-40% wt).

**Table 1:** Summary of general characteristics of studies and intervention.

Study ID	Country	Study design	Sample size	Type of S-PRG	Group	Composition	Comparison/Control material class
(Kamijo et al. [38])	Japan	In vitro	5 rectangular (64 × 10 × 3.3 mm)/group	Resin based S-PRG	S-PRG (Shofu Inc. Kyoto, Japan)	NA	Resin-based filler control (non-S-PRG)
(Wang et al. [37])	China	In vitro	4 disc/group	Beauti Sealnat	S-PRG containing fissure sealants	TEGDMA, UDMA, S-PRG fillers	Resin sealant/composite sealant (Delton), glass ionomer-based sealant (Teethmate F1-2.0), glass ionomer cement sealant, and non-S-PRG control
(Kaga et al. [31])	Japan	In vitro	6 disc (6 × 3 mm)/group	S-PRG (Shofu, Kyoto, Japan)	S-PRG (Lot no. SIR-20901) containing fissure sealants	TEGDMA, UDMA, S-PRG fillers	Resin sealant/composite sealant (Delton), glass ionomer-based sealant (Teethmate F1-2.0), and glass ionomer cement sealant
(Shimazu et al. [53])	Japan	In vitro	5/group	BeautiSealant (Shofu Inc., Kyoto, Japan)	Resin based S-PRG	S-PRG filler (40% wt), TEGDMA, UDMA	Glass ionomer-based sealant (Teethmate F-12.0) and resin sealant/composite sealant (Delton FS + )
(Zafar [34])	Saudi Arabia	In vitro	6 disc (6 × 2 mm)/group	Beautifil	Giomer	Bis-GMA/TEGDMA, Glass fillers, catalyst, S-PRG fillers (Shofu Inc., Kyoto, Japan)	Resin-modified glass ionomer cement (Fuji II LC)
(Suzuki et al. [39])	Japan	In vitro	Disc (15 × 1 mm)	Beautifil II (Shofu Inc. Kyoto, Japan)	S-PRG	Phosphoric acid monomer, TEGDMA, methacrylic acid monomer, carboxylic acid monomer, bis-MPEPP, photoinitiator	Glass ionomer cement (Fuji VII)
(Yassen et al. [33])	USA	In vitro	3 Disc (4 × 2 mm)/group	S-PRG root cement (Lot No. 14315, Shofu Inc. Kyoto, Japan)	S-PRG filler containing root repair cement was provided by Shofu	NA	Intermediate restorative material (IRM) and calcium silicate cement/mineral trioxide aggregate (MTA)
(Kiatsirirote et al. [23])	USA	RCT	110 (Age:49.7, 24 M: 86 F)	Modified Resin Denture Containing S-PRG Fillers (particle size 4.1 μm, Lot 140122, Shofu Inc. Kyoto, Japan)	Resin-based S-PRG	URBAN RESIN containing white S-PRG filler	Resin denture base control without S-PRG
(Burtea et al. [35])	Romania	In vitro	5 disc/group	Beautifil II-shade A30, batch number 051215, (PN1420 2015-04), Shofu Inc., Kyoto, Japan	Giomer	Aqueous solution (50%) of ternary copolymer P (AA-co-IA-co-Leu), itaconic acid (IA), and N-acryloyl-L-leucine (Leu) (molar ratio between AA/IA/Leu being 4:1:0.5) with the superficially active glass powder G having the oxidic composition SiO_2_ (49%), Al_2_O_3_ (22%), CaF_2_ (29%), in a weight ratio of 1/2.4	Resin-based filler controls (non-S-PRG)
(Şirinoğlu-çapan et al. [54])	Turkey	In vitro	60 Cylindrical (5.0 mm diameter × 2.0 mm height)/group	Beautifil II (Shofu, Japan)	Giomer	TEGDMA, Bis-GMA, S-PRG filler, Aluminofluoroborosilicate glass, DL-camphorquinone	Resin-modified glass ionomer cement (Fuji II LC Capsule), conventional glass ionomer cement (Fuji IX GP Capsule), compomer (Dyract XP), glass hybrid restorative material (GCP Glass Fill), and resin composite (Filtek Z250)
(Shimizu et al. [32])	Japan	In vitro	10/group	Beautifil Flow Plus F03 and SI-R21701 F10 (Lot no. F10101718N3, Shofu Inc., Kyoto, Japan),	S-PRG	UDMA, Phosphonic acid monomer, HEMA, photoinitiator, S-PRG filler based on fluoroboro aluminosilicate glass, Glass powder, Silica and Bis-GMA, TEGDMA, Reaction initiator, S-PRG filler based on fluoroboro-aluminosilicate glass	Self-adhesive flowable resin composite (Fusio Liquid Dentin) and glass ionomer cement (FX ULTRA)
(Bergantin et al. [41])	Brazil	In vitro	10 block/group	Beautifil II Lot 051829/Color A2 (Shofu Inc., Kyoto, Japan)	S-PRG-based composite resin	TEGDMA, Bis-GMA, multifunctional filler, S-PRG filler based on F-Br-Al-Si glass	Conventional resin composite (CR), bulk-fill resin composite (BF), resin-modified glass ionomer cement (RMGI), and glass hybrid restorative system (GHRS)
(Kim [40])	S. Korea	In vitro	16/group	Beautifil Flow Plus (Shofu, Kyoto, Japan)	S-PRG filler, Aluminofluoro-borosilicate glass (47%)	TEGDMA, Bis-GMA, Initiator (53%)	Resin-modified glass ionomer cement (Fuji II LC) and alkasite restorative material (Cention N)
(Pimentel et al. [42])	Brazil	In vitro	12 Disc (6 × 2)/group	Beautifil II (Batch: 012079, particle size: 0.8 μm)	Nanohybrid with S-PRG particles	Bis-GMA, glycol dimethyl ether, triethylene aluminofluoro-borosilicate glass, aluminum oxide, DL-camphorquinone, and others	Conventional nanohybrid resin composite (Forma)
(Tsuji et al., 2024)	Japan	In vitro	4 for ion concentration and 12 for flexural strength cone shaped (10 × 15 × 1 mm)/group	Experimental light-curing resin containing Shofu S-PRG filler	Resin based S-PRG (30% wt)	Urethane dimethaceylate, Photoinitiator, Polyfunctional methacrylate, Others, Ultra-fine filler, S-PRG filler	Self-curing acrylic resin (Unifast III) and resin without S-PRG filler (0 wt%)
(Ubolsa-Ard et al. [14])	Thailand	In vitro	8 disc (10×2 mm)/group	Resin-based S-PRG	S-PRG (Shofu Inc., Kyoto, Japan)	UDMA, HEMA, S-PRG fillers (30% wt)	Demineralized dentin control substrate
(Kaga et al. [30])	Japan	In vitro	10/group	Auto-polymerizing resin with S-PRG	S-PRG variants (particle sizes 0.8–4.1 μm)	MMA, PMA, S-PRG fillers	Auto-polymerizing resin without S-PRG
(Islam et al. [22])	Australia	In vitro	12/group	Repair composite with S-PRG	S-PRG (Shofu Inc., Kyoto, Japan)	Bis-GMA, TEGDMA, S-PRG (20% wt)	Conventional resin composite

### Characteristics of outcomes assessment

Fluoride release and recharging are commonly measured using ion-selective electrodes [23, 31–35], ion meters [36, 37], and immersion methods for recharging [38]. Most procedures were performed at 37 °C under varying pH conditions (Table 2).Furthermore, the diametral tensile strength [31], shear bond strength [39, 40], flexural strength [35, 36, 38], and wear/erosion [41, 42] of these S-PRGs were evaluated using various techniques and methods, as shown in Table 2.

**Table 2:** Summary of fluoride and strength assessment methods used for S-PRG.

Study ID	Fluoride release/recharge assessment method	Quantitatively pooled in meta-analysis	Temp.	PH	Mechanical properties (Diametral tensile strength/Flexural Strength/Wear/erosion) assessment	Conclusion
(Kamijo et al. [38])	Specimens immersion in 3 ml (9,000 ppm) fluoride solution for 8 hours to recharge	No	37	NA	Flexural strength measured according to ISO 1567	S-PRG filler has great recharge and release capabilities
(Wang et al. [37])	Ion meter	Yes	37	4, 7, 10	NA	S-PRG sealants showed lower fluoride release than some comparator materials under the tested conditions
(Kaga et al. [31])	Ion selective electrode connected to ion analyzer	Yes	37	NA	DTS was measured by taking diameter, thickness, and power at failure	S-PRG had DTS and more fluoride releasing power
(Shimazu et al. [53])	Ion concentration was measured daily	Yes	NA	NA	NA	S-PRG sealant release significant (*p* < 0.05) amount of fluoride
(Zafar [34])	Ion selective electrode	Yes	37	NA	NA	S S-PRG released significantly lower fluoride than Fuji II LC (*p* < 0.05)
(Suzuki et al. [39])	Inductively coupled plasma atomic emission spectroscopy	No	37	4	Shear bond strength testing using a universal testing machine	S-PRG filler showed greater fluoride release under the tested conditions
(Yassen et al. [33])	Ion-specific electrode connected to an ion meter	No	37	4, 7, 10	NA	S-PRG cement demonstrated multiple ion releasing abilities and low bonding strength
(Kiatsirirote et al. [23])	Ion-selective electrode direct method	No	NA	NA	NA	The resin denture base containing S-PRG fillers (20% wt) demonstrated an initial fluoride release that increased saliva fluoride concentrations
(Burtea et al. [35])	Ion selective electrode	Yes	37	NA	The flexural strength was determined according to ISO 4049 and 2000	Experimental fillers showed comparable or greater performance than commercial S-PRG under the tested conditions
(Şirinoğlu-çapan et al. [54])	NA	No	37	5, 7	NA	Fluoride-release from glass ionomer-based materials was higher than S-PRG
(Shimizu et al. [32])	Fluoride ion electrode method	Yes	37	NA	NA	The S-PRG filler containing self-adhesive flowable resin composites had higher fluoride release
(Bergantin et al. [41])	NA	No	25	2.5	Wear/erosion: Abrasion using a toothbrush machine with fluoridated dentifrice: water slurry (3:1) for 60 seconds	S-PRG-based-composites can diminish surrounding enamel loss only against erosion alone
(Kim [40])	NA	No	37	4, 7, 10	Universal testing machine at a crosshead speed of 1 mm/min until bond failure occurred	Non-significant differences (*p* = 0.51) in the bond strength among S-PRG and others
(Pimentel et al. [42])	NA	No	37	2–2.6	Wear/erosion: Samples were immersed in a remineralizing solution during the experiment	Hydrochloric acid: Non-significant (*p* > 0.05) increase in the roughness of both composites. Citric acid: The resin composite with S-PRG filler showed a significant (*p* = 0.003) change in roughness
(Tsuji et al., 2024)	Inductively coupled plasma and an ion meter	Yes	37	NA	Flexural strength tests were performed according to the ISO 4049 and 1567	The findings suggest that this S-PRG-containing resin may be suitable for provisional restoration
(Ubolsa-Ard et al. [14])	Ion-selective electrode	No	37	7	Ultimate tensile strength via microtensile testing	S-PRG enhanced dentin remineralization and tensile strength by 18% (*p* < 0.05)
(Kaga et al. [30])	NA	No	37	NA	Flexural strength per ISO 4049	Optimal S-PRG particle size (2.5 μm) increased flexural strength by 15% (*p* < 0.01)
(Islam et al. [22])	NA	No	37	NA	Shear bond strength post-mechanical alteration	S-PRG improved repair bond strength by 20% (*p* = 0.01)

Footnote : Only studies with sufficiently compatible quantitative fluoride-release data and extractable summary statistics were included in the meta-analysis; the remaining studies were narratively synthesized.

*ISO* International Organization for Standardization, *DTS*Direct Tensile Strength, *S-PRG* Surface-Pre Reacted Glass, *ppm* Parts Per Million, *NA* Not Available.

### Outcomes

#### Meta-analyses (fluoride release)

**Fig. 2:**
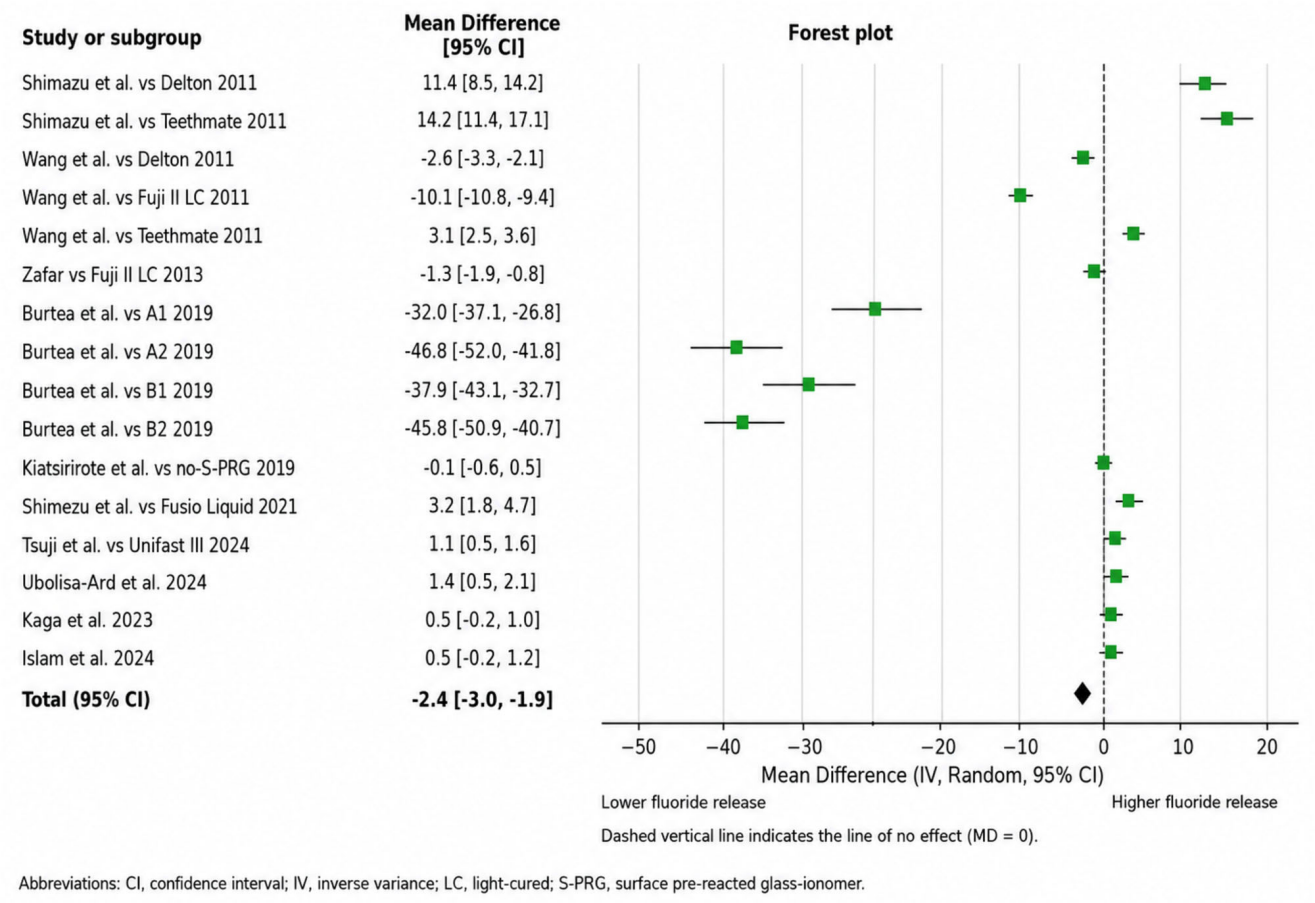
Forest plot of fluoride-release outcomes comparing S-PRG-containing materials with non-S-PRG comparator materials. Comparator groups included different restorative material classes, such as resin-based materials and glass ionomer-based materials; therefore, the pooled estimate should be interpreted as an exploratory overall comparison.

Seven studies provided quantitative fluoride-release data that were eligible for meta-analysis. The comparator materials varied widely between studies and comprised both fluoride-releasing glass ionomer-based materials and less bioactive resin-based materials. A statistically significant overall difference between S-PRG-containing materials and comparators was found in the pooled effect estimate (mean difference: –3.21; 95% CI: –4.12 to –2.30; *p* < 0.01). However, there was high heterogeneity (I² = 98%). The extracted summary statistics for studies contributing to the widest confidence intervals were rechecked against the source articles, and no data entry discrepancies were identified. The broad confidence intervals of some comparisons were likely due to the small sample sizes and considerable within-study variability. As such, this pooled estimate should be viewed as an exploratory overview rather than a homogeneous effect for all comparator classes or test conditions. The forest plot of fluoride release is shown in Fig. 2. Overall, the certainty of the evidence was considered low because of inconsistency (Table 4).

#### Mechanical strength (sub-group analyses)

**Fig. 3:**
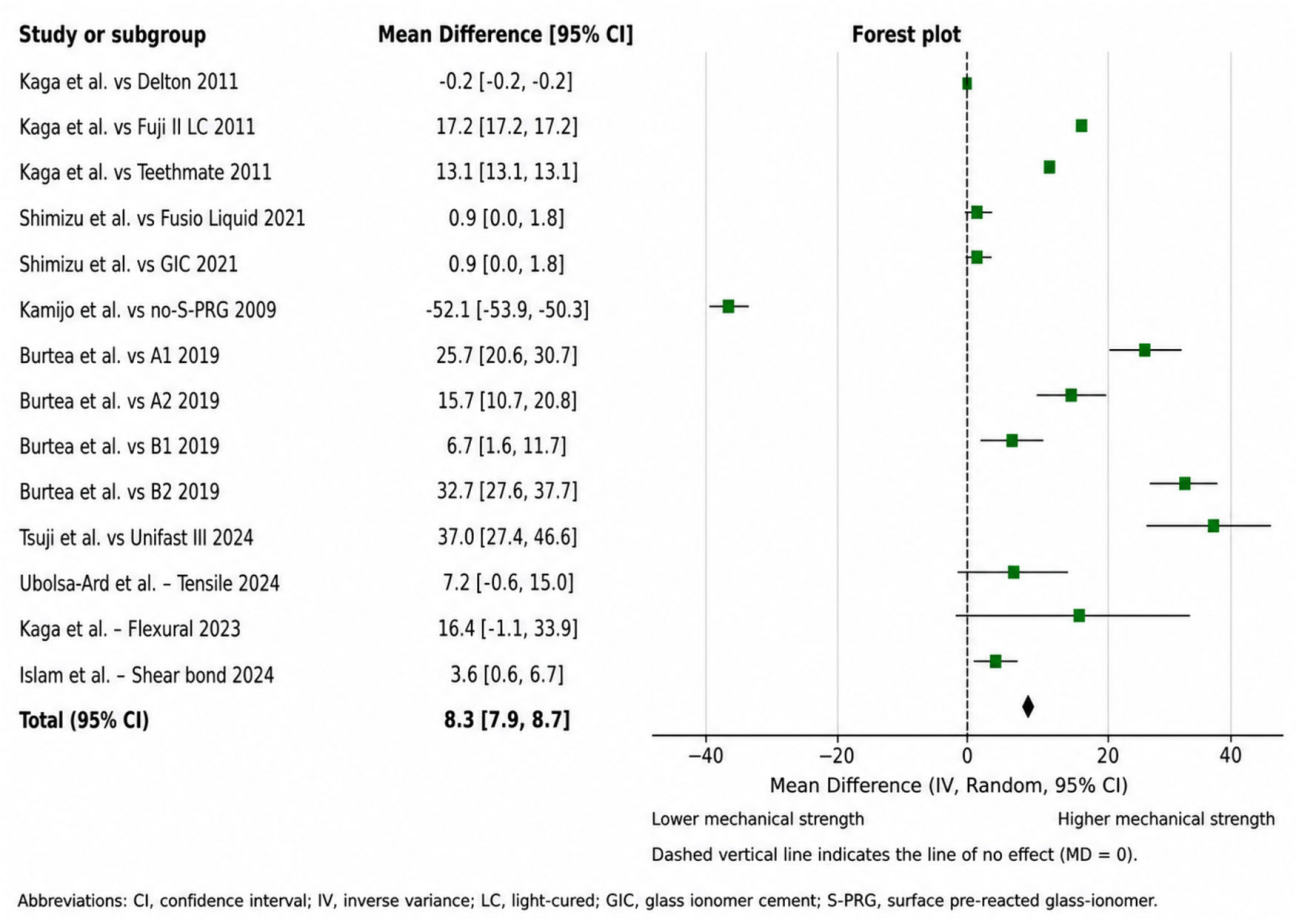
Forest plot of mechanical-property outcomes comparing S-PRG-containing materials with comparator materials, including subgroup analyses for diametral tensile strength (DTS) and flexural strength. Because several studies had small sample sizes and substantial variability, some confidence intervals were broad, and the pooled estimates should be interpreted cautiously.

Subgroup analyses were performed for mechanical properties, including diametral tensile strength (DTS) and flexural strength. The pooled estimate across studies that reported DTS was 6.50 (95% CI: –0.35 to 13.35; I² = 98%), and the pooled estimate for flexural strength was 11.20 (95% CI: –28.50 to 50.90; I² = 99%). The overall pooled estimate for mechanical properties was 9.45 (95% CI: –7.89 to 26.79; I² = 99%). Wide confidence intervals for some studies may be due to the limited sample sizes and large variability in the observed outcomes. Despite these differences, the confidence intervals were wide, and heterogeneity was high. This shows that S-PRG-containing materials were not superior in all the situations tested. Therefore, we expect these results to be interpreted with caution. A forest plot depicting the results for the mechanical properties is shown in Fig. 3. The level of certainty of the evidence was low (Table 4).

### Methodological quality assessment

Most in vitro studies were judged to have either a medium or low risk of bias according to the QUIN criteria, as shown in Table 3. Two domains were not applicable to this study: operator details and outcome assessor details. However, most studies had zero scores in the sample size calculation domain, except for two studies [40, 41]. Similarly, in the randomization domain, all studies had zero scores, except for four studies, which had a score [33, 40–42], as indicated in Table 3. One study was an RCT, which was also found to have a low risk of bias [23].

**Table 3:** Quality assessment of included studies.

Studies	Questions											Total points	% age	Status
	Aims/objectives	Sample size calculation	comparison group	Methodology explanation	Operator details	Randomization	Method of measurement of outcome	Outcome assessor details	Blinding	Statistical analysis	Presentation of results			
(Kamijo et al. [38])	2	0	2	2	NA	0	2	NA	0	2	2	12	66.6	M
(Wang et al. [37])	2	0	2	2	NA	0	2	NA	0	2	2	12	66.6	M
(Kaga et al., 2011)	2	0	2	2	NA	0	2	NA	0	2	2	12	66.6	M
(Shimazu et al. [53])	2	0	2	2	NA	0	2	NA	0	2	2	12	66.6	M
(Zafar [34])	2	0	2	2	NA	0	2	NA	0	2	2	12	66.6	M
(Suzuki et al. [39])	2	0	2	2	NA	0	2	NA	0	2	2	12	66.6	M
(Yassen et al. [33])	2	0	2	2	NA	1	2	NA	0	2	2	13	72.2	L
(Burtea et al. [35])	2	0	2	2	NA	0	2	NA	0	2	2	12	66.6	M
(Şirinoğlu-çapan et al. [54])	2	0	2	2	NA	0	2	NA	0	2	2	12	66.6	M
(Shimizu et al. [32])	2	0	2	2	NA	0	2	NA	0	2	2	12	66.6	M
(Bergantin et al. [41])	2	2	2	2	NA	1	2	NA	0	2	2	15	83.3	L
(Kim [40])	2	2	2	2	NA	1	2	NA	0	2	2	15	83.3	L
(Pimentel et al. [42])	2	0	2	2	NA	1	2	NA	0	2	2	13	72.2	L
(Tsuji et al., 2024)	2	0	2	2	NA	0	2	NA	0	2	2	12	66.6	M
(Ubolsa-Ard et al. [14])	2	0	2	2	NA	0	2	NA	0	2	2	12	66.6	M
(Kaga et al., 2023)	2	2	2	2	NA	1	2	NA	0	2	2	15	83.3	L
(Islam et al. [22])	2	0	2	2	NA	0	2	NA	0	2	2	12	66.6	M

*L*low, *M*medium, *H*high, *NA*not available.

### GRADE assessment

Table 4 presents the two main outcomes: fluoride release and mechanical strength (DTS and flexural strength). Both outcomes showed overall differences between S-PRG-containing materials and comparator groups; however, the certainty of the evidence remained low because of marked inconsistency and the predominance of in vitro evidence. However, owing to the high heterogeneity of outcomes, there is a low certainty of evidence. Despite the lower evidence certainty, this suggests the potential use of S-PRG in dental restorations.

**Table 4:** GRADE assessment of the outcomes.

Balancing bioactivity and strength: a systematic review of fluoride release and mechanical properties of S-PRG fillers in dental materials					
Population	Samples for dental restoration				
Intervention	S-PRG				
Control	Control group without use of S-PRG				
Outcomes	Relative effect (95% CI), studies/sample size	With S-PRG	Without S-PRG	Certainty of evidence (GRADE)	Key message
Fluoride release	–3.54 (95% CI; –4.57 to –2.50), 8/132	Increased fluoride	Reduced fluoride	ƟƟ (Low)	Due to high heterogeneity, outcomes should be used with caution
Mechanical strength	8.91 (95% CI; –9.13 to 26.96), 6/80	Increased strength	Reduced strength	ƟƟ (Low)	

## Discussion

For restorative dental materials, sustained fluoride release and adequate mechanical performance are desirable properties, particularly for bioactive materials such as S-PRG. Therefore, this systematic review and meta-analysis was designed to evaluate the fluoride ion-releasing capabilities and mechanical properties of S-PRG ionomer fillers used in dental restoration.

In the present study, a statistically significant (*p* < 0.01) impact of S-PRG ionomer fillers was observed in the release fluoride ions compared with other materials that did not contain S-PRG ionomers. However, high heterogeneity was also observed, which might be due to the comparison of S-PRG ionomers with multiple alternative materials in the same study. Another reason might be the small sample size, which was also responsible for the heterogeneity. Our findings are in agreement with those of other in vitro studies, which concluded that despite the initial giomer (S-PRG ionomer) with no initial fluoride burst, over time, it released a higher amount of fluoride than Fuji II and other materials [43]. Similarly, in another study, a higher amount of fluoride ions was observed in commercially available S-PRG material (Reactmer) than in composites (Xeno CF) and compomer (Dyract) [44]. In contrast, another comparative study reported that Beautifil II, a commercial S-PRG restorative material, exhibited lower fluoride release than some non-S-PRG comparators, including glass ionomer-based materials [45]. Similarly, the outcomes of other studies were also different from our outcomes; they found that conventional GICs had better and higher fluoride-releasing ability than S-PRG (giomers) [46]. This variation in the outcomes of different studies might have occurred due to the material type, their differential chemical composition, powder-to-liquid ratio during the preparation of materials, surface area, shape of the material, mixing techniques, storage medium, storage environment (temperature and pH), and finishing procedures that affect the fluoride-releasing ability of dental restoration materials [47]. These methodological differences, together with the small sample sizes in several studies, may also explain the broad confidence intervals observed in pooled analyses. Furthermore, the extent of the hydrogel matrix is also considered another important factor that has a significant impact on the release of fluoride ions [47].

The most likely explanation for this heterogeneity is the difference in the class of comparator material. While some studies directly compared S-PRG to conventional resin-based materials with limited fluoride release potential, other studies compared S-PRG with glass ionomer-based materials, which are fluoride-releasing chemically active materials. Because these materials differ substantially in composition, ion release profiles, and baseline mechanical properties, a single pooled estimate may obscure clinically important differences between the two comparison groups. This also accounts for why some studies included in this analysis favored S-PRG against resin-based controls while other studies favored glass ionomer-based materials for fluoride release.

Pooled estimates of mechanical properties indicated differences between S-PRG-containing materials and comparators. Nonetheless, these analyses were associated with wide confidence intervals as well as extreme heterogeneity. As such, the evidence does not support a consistent finding of mechanical superiority in all contexts. Individual studies noted increased flexural strength or other measures of performance when using S-PRG materials in comparison with selected comparator material [48]. yet did not demonstrate consistent results across all study types and material comparisons.

Similarly, another in vitro study compared Beautifil, an S-PRG-based giomer, with Fuji II, F2000, Alpha-Dent, and Solare Anterior, and reported significantly higher flexural strength for Beautifil than for Fuji II, Alpha-Dent, and F2000 (*p* < 0.05) [49]. Moreover, the flexural strength of S-PRG also increased with the content, as at 10 wt %, its flexural strength was 62.14 MPa, and at 20 wt% it became 68.68 MPa. Thus, the content and particle size significantly affected the flexural strength of S-PRG [30]. For instance, another study reported that the addition of 5 wt% S-PRG to ceramic fillers increased flexural strength by 15.8%, DTS by 25%, and wear resistance by 9.6% [50]. Notably, all restoration materials should have high flexural strength, which helps to enhance the longevity of restorations. Thus, according to ISO 4049, the minimum requirement for dental restoration flexural strength is 80 MPa [51, 52]. The present pooled findings should not be interpreted as confirming that all S-PRG-containing materials consistently meet or exceed comparator performance under all laboratory or clinical conditions.

Our study has various strengths and limitations, including a comprehensive review of the available literature and its analysis. In addition, inter-reviewer agreement was not quantified using a kappa statistic, although independent duplicate screening and consensus resolution were performed. In addition, it also highlights the fluoride-releasing ability of S-PRG fillers used in dental restorations. However, certain limitations of this study must be highlighted. For instance, high heterogeneity was observed among the studies, which may be due to the small sample size, multiple comparisons from the same study, chemical composition, storage conditions (temperature and pH), and mixing techniques used for preparation of S-PRG fillers. Some studies showed broad confidence intervals because of small sample sizes, variability in outcome dispersion, and differences in comparator materials and testing protocols, rather than identifiable data-entry errors. Therefore, the results of this study should be interpreted with caution. Furthermore, subgroup analyses of the type of material, different weights of S-PRG (%), and storage conditions could not be performed because of the unavailability of the required data. Most of the included evidence was derived from in vitro studies (16/18), which allowed for controlled assessment of fluoride release and mechanical properties but limited direct clinical extrapolation. In vivo studies, such as those by Kiatsirirote et al. [23] and Miyaji et al. [20], are limited (2/18 studies) due to ethical concerns (e.g., invasive testing), high costs, and the prioritization of in vitro testing for early material development. Although in vitro findings provide useful preliminary information, they may not fully translate to clinical settings because of dynamic oral conditions, such as pH fluctuations, microbial activity, salivary effects, and masticatory loading.

## Conclusions

The present systematic review and meta-analysis indicated that S-PRG-containing materials could offer fluoride release potential and may differ in selected mechanical properties, such as DTS and flexural strength. Overall, the fluoride-release behavior and mechanical properties of S-PRG-containing materials tended to differ from those of the comparator materials; however, the direction varied based on the comparator type and conditions. While the evidence available for mechanical properties showed differences when analyzed in pooled analyses, it was not consistent with S-PRG superiority in all tested conditions. Further studies with high heterogeneity should be interpreted cautiously. In addition, since most of the included evidence came from in vitro studies, its clinical extrapolation is limited. Future investigations should implement standardized fluoride-release measurement protocols, uniform mechanical testing methods, and well-constructed controlled clinical trials.

## Supplementary information

Supplementary table 1

## Data Availability

The data supporting the findings of this study are available from the corresponding author upon reasonable request.
