# Supplementary material for: Fluoride release and mechanical properties of S-PRG fillers in dental materials: a systematic review and meta-analysis
Source: BDJ Open. 2026 May 15;12:52. doi: 10.1038/s41405-026-00442-z (PMC13179356; doi:10.1038/s41405-026-00442-z)
Supplement: Supplementary file 1 — Supplementary table 1 [file 41405_2026_442_MOESM1_ESM.docx]

**Supplementary table 1.** Literature search from different databases.

| **PubMed**   1. ("surface pre-reacted glass-ionomer filler"[All Fields] OR "S-PRG fillers"[All Fields]) AND ("fluoride release"[All Fields] OR "fluoride delivery system"[All Fields]) 2. ("surface pre-reacted glass-ionomer filler"[All Fields] OR "S-PRG fillers"[All Fields]) AND ("mechanical properties"[All Fields] OR "strength"[All Fields] OR "flexural strength"[All Fields] OR "durability"[All Fields] OR "wear resistance"[All Fields]) 3. ("surface pre-reacted glass-ionomer filler"[All Fields] OR "S-PRG fillers"[All Fields]) AND "Dentistry"[All Fields] 4. ("surface pre-reacted glass-ionomer filler"[All Fields] OR "S-PRG fillers"[All Fields]) AND ("fluoride release"[All Fields] OR "fluoride delivery system"[All Fields]) AND ("mechanical properties"[All Fields] OR "strength"[All Fields] OR "flexural strength"[All Fields] OR "durability"[All Fields] OR "wear resistance"[All Fields]) AND "Dentistry"[All Fields] |
| --- |
| **ScienceDirect**  (“surface pre-reacted glass-ionomer filler” OR “S-PRG fillers”) AND (“fluoride release” OR “fluoride delivery system”) AND (“mechanical properties” OR “strength” OR “flexural strength”) AND (“Dentistry”) |
| **Scopus**  (“surface pre-reacted glass-ionomer filler” OR “S-PRG fillers”) AND (“dental restoration materials” OR “dental fillings” OR “Dentistry”) |
| **Google Scholar**  (“surface pre-reacted glass-ionomer filler” OR “S-PRG fillers”) AND (“fluoride release” OR “fluoride delivery system”) AND (“mechanical properties” OR “strength” OR “flexural strength”) AND (“Dentistry”) |
